# Concurrent Expression of *Oct4* and *Nanog* Maintains Mesenchymal Stem-Like Property of Human Dental Pulp Cells

**DOI:** 10.3390/ijms151018623

**Published:** 2014-10-15

**Authors:** Chuan-En Huang, Fang-Wei Hu, Chuan-Hang Yu, Lo-Lin Tsai, Tzu-Hsin Lee, Ming-Yung Chou, Cheng-Chia Yu

**Affiliations:** 1 School of Dentistry, Chung Shan Medical University, No. 110, Sec. 1, Jianguo N. Rd., Taichung 40201, Taiwan; E-Mails: csmulab1@gmail.com (C.-E.H.); fang0989909009@gmail.com (F.-W.H.); d94422004@ntu.edu.tw (C.-H.Y.); tsailolin@mail2000.com.tw (L.-L.T.); cherylee@csmu.edu.tw (T.-H.L.); 2 Department of Dentistry, Chung Shan Medical University Hospital, No. 110, Sec. 1, Jianguo N. Rd., Taichung 40201, Taiwan; 3 Institute of Oral Sciences, Chung Shan Medical University, No. 110, Sec. 1, Jianguo N. Rd., Taichung 40201, Taiwan

**Keywords:** dental pulp stem cells, *Oct4*, *Nanog*

## Abstract

Human dental pulp stem cells (DPSCs), unique mesenchymal stem cells (MSCs) type, exhibit the characteristics of self-renewal and multi-lineage differentiation capacity. *Oct4* and *Nanog* are pluripotent genes. The aim of this study was to determine the physiological functions of *Oct4* and *Nanog* expression in DPSCs. Herein, we determined the critical role of an *Oct4*/*Nanog* axis modulating MSCs properties of DPSCs by lentiviral-mediated co-overexpression or co-knockdown of *Oct4*/*Nanog* in DPSCs. MSCs properties including osteogenic/chondrogenic/adipogenic induction differentiation was assayed for expression of osteogenic/chondrogenic/adipogenic markers by quantitative real-time RT-PCR analysis. Initially, we observed that the expression profile of *Oct4* and *Nanog* in dental pulp cells, which exerted properties of MSCs, was significantly up-regulated compared to that of STRO-1^−^CD146^−^ dental pulp cells. Down-regulation of *Oct4* and *Nanog* co-expression significantly reduced the cell proliferation, osteogenic differentiation capability, STRO-1, CD146, and Alkaline phosphatase (ALP) activity of DPSCs. In contrast, co-overexpression of *Oct4* and *Nanog* enhanced the expression level of STRO-1 and CD146, proliferation rate and osteogenic/chondrogenic/adipogenic induction differentiation capability, and expression of osteogenic/chondrogenic/adipogenic induction differentiation markers. Our results suggest that *Oct4*-*Nanog* signaling is a regulatory switch to maintain properties in DPSCs.

## 1. Introduction

Dental pulp stem cells (DPSCs) are a recently developed technology that holds promise for stem cell biology and regenerative medicine. DPSCs are well known to be multi-potent cells capable of differentiation along multiple lineages [1]. DPSCs have the remarkable potential for multi-lineage differentiation capacity including osteoblast [2], cartilage [3], adipocyte [4], muscle [5], hepatocyte [6], and neurons [7]. Therefore, an improved comprehension of the cellular and molecular mechanisms, which modulate self-renewal and differentiation properties of DPSCs, could be pursued to bring forth future progress in regenerative medicine.

*Oct4* along with *Nanog* is part of the key set of transcription factors that are involved in the maintenance of pluripotency and self-renewal in undifferentiated embryonic stem cells (ESCs) [8]. Hyslop and colleagues reported that down-regulation of *Nanog* in human ESCs induces pro-extraembryonic lineage differentiation, evidenced by the up-regulated endoderm- and trophectoderm-associated genes, suggesting that *Nanog* acts as a gatekeeper of pluri-potency in human embryonic development [9]. The leukemia inhibitory factor (LIF) has been utilized to maintain the symmetrical self-renewal of mouse ESCs [10]. Constitutively activated *Oct4* from an exogenous promoter in ESCs still required LIF for inducing self-renewal in ESCs [11]. *Nanog* overexpression relieves ESCs self-renewal from dependence on the activity of the leukemia inhibitory factor-signal transducer and activator of transcription 3 (LIF-STAT3) pathway. Moreover, Chambers’ report showed that *Nanog* is expressed in *Oct4*-deficient embryos, and *Nanog* overexpression does not revert the differentiation program of ESCs triggered by *Oct4* down-regulation [12]. These results suggest that Nanog is not just a downstream version of *Oct4* and, *Nanog* and *Oct4* work in concert to support stem cell potency and self-renewal. *Oct4* and *Nanog*, are shown to reprogram human somatic fibroblasts into embryonic stem cell-like pluripotent cells, termed inducible pluripotent stem cells [13]. Nevertheless, *Oct4*/*Nanog* mediated molecular mechanisms in regulating DPSCs are still unclear.

The detailed molecular mechanisms involved in the regulatory links between *Oct4*/*Nanog* and DPSCs properties are still poorly understood. Herein, we demonstrate a critical role of *Oct4*/*Nanog* overexpression in the enhancement of proliferation rate and promoting osteogenic/chondrogenic/adipogenic induction differentiation of DPCs. Additionally, down-regulation of *Oct4*/*Nano*g lessens MSCs properties of DPSCs. Ultimately, we demonstrated the functional roles of *Oct4*/*Nanog* co-expression in DPSCs.

## 2. Results

### 2.1. Increased Expression of Oct4 and Nanog Expression in STRO-1^+^CD146^+^ DPSCs (Dental Pulp Stem Cells)

**Figure 1:**
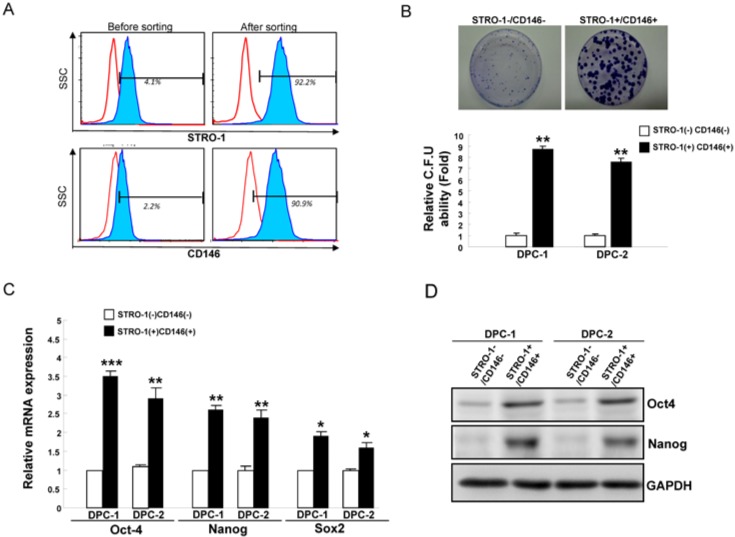
Enriched *Oct4* and *Nanog* expression in STRO-1^+^CD146^+^ DPSCs (Dental Pulp Stem Cells). (**A**) The expression of STRO-1 and CD146 in dental pulp cells was analyzed by flow cytometry; (**B**) To elucidate the capabilities of colony formation of STRO-1^−^CD146^−^ and STRO-1^+^CD146^+^ dental pulp cells, single-cell suspensions of dental pulp cells were plated and analyzed as described in the experimental section; *Oct4*, *Nanog*, and Sox2 mRNA expression in STRO-1^+^CD146^+^ and STRO-1^−^CD146^−^ dental pulp cells were examined by real-time RT-PCR analysis (**C**) and western blotting analysis (**D**). The amount of GAPDH protein of different crude cell extracts was referred to as a loading control. Error bars correspond to SD. Data shown here are the mean ± SD of three independent experiments. (* *p* < 0.05; ** *p* < 0.01; *** *p* < 0.001).

The STRO-1^+^ and CD146^+^ dental pulp cells (DPCs) have been shown to exhibit MSCs properties and these markers have been used to identify dental pulp stem cells (DPSCs) [14]. We isolated STRO-1^+^CD146^+^ primary DPSCs from human dental pulp human tissues by using a Flow-activated cell sorting (FACS) cell sorter (Figure 1A). We next performed a colony-forming assay to evaluate the colony-forming efficiency of STRO-1^+^CD146^+^ and STRO-1^−^CD146^−^ DPCs, respectively. Apparently, the colony-forming efficiency of STRO-1^+^CD146^+^ cells was significantly higher than that of the STRO-1^−^CD146^−^ cells (Figure 1B). By using quantitative real-time RT-PCR, we observed increased expression of ESCs-related stemness genes, especially *Oct4* and *Nanog*, in STRO-1^+^CD146^+^ cells (Figure 1C). As shown in Figure 1D, *Oct4* and *Nanog* protein expression was readily detectable in Strol^+^CD146^+^ but was low or undetectable in STRO-1^−^CD146^−^ cells. Together, we hypothesized that up-regulation of Oct4 and Nanog might be crucial for modulating MSCs characteristics of DPCs.

### 2.2. Silencing Oct4 or Nanog Expression Did not Affect the Proliferation Rate and ALP (Alkaline Phosphatase) Activity in STRO-1^+^CD146^+^ DPSCs

**Figure 2:**
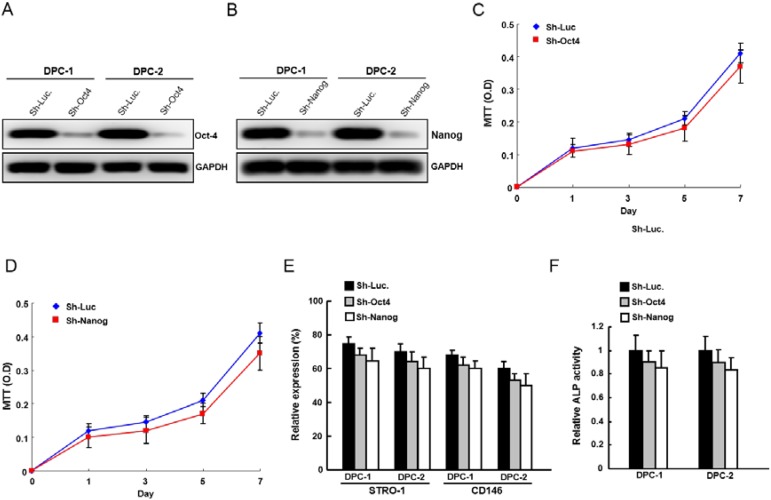
Depletion of *Oct4* or *Nanog* expression did not affect proliferation rate and ALP activity in STRO-1^+^CD146^+^ DPSCs. The silencing effect of *Oct4* (**A**) or *Nanog* (**B**) shRNA in DPSCs was validated translationally by western blotting. Immunoblotting against anti-Oct4, anti-Nanog, or anti-GAPDH antibodies was performed as indicated; The amount of GAPDH protein of different crude cell extracts was referred to as loading control and for further quantification; The proliferation rate of sh-*Oct4* (**C**) or sh-*Nanog* (**D**) in STRO-1^+^CD146^+^ DPSCs was analyzed by a 3-(4,5-dimethylthiazol-2-yl) 2,5-diphenyl tetrazolium bromide (MTT) assay; (**E**) The expression of STRO-1 and CD146 of *Oct4* or *Nanog*-knockdown DPSCs was analyzed by flow cytometry analysis; (**F**) ALP activity of *Oct4* or *Nanog* knockdown DPSCs was analyzed. Data shown here are the mean ± SD of three independent experiments.

To investigate whether *Oct4* or *Nanog* plays a role in maintaining MSCs properties of STRO-1^+^CD146^+^ DPSCs, the approach of loss-of-function of *Oct4* or *Nanog* expression was first conducted. Down-regulation of *Oct4* or *Nanog* expression in DPSCs was achieved by viral transduction with lentiviral vector expressing small hairpin RNA (shRNA) targeting *Oct4* and *Nanog* and lentiviral vector expressing shRNA against luciferase (sh-Luc) used as a control. Immunoblotting analyses confirmed that lentivirus expressing sh-*Oct4* or sh-*Nanog* markedly reduced the expression level of *Oct4* (Figure 2A) or *Nanog* (Figure 2B) protein in transduced STRO-1^+^CD146^+^ DPSCs. However, single silencing *Oct4* (Figure 2C) or *Nanog* (Figure 2D) expression did not affect the proliferation rate of STRO-1^+^CD146^+^ DPSCs. DPSCs infected with sh-*Oct4* or sh-*Nanog* expressing lentivirus did not change the expression level of STRO-1 and CD146 in DPSCs (Figure 2E). The ALP activity in DPSCs did not change in sh-*Oct4* or sh-*Nanog* knockdown DPSCs (Figure 2F).

### 2.3. Down-Regulation of Oct4 and Nanog co-Expression Reduces the Proliferation Rate and Odontogenic/Osteogenic Properties in STRO-1^+^CD146^+^ DPSCs

**Figure 3:**
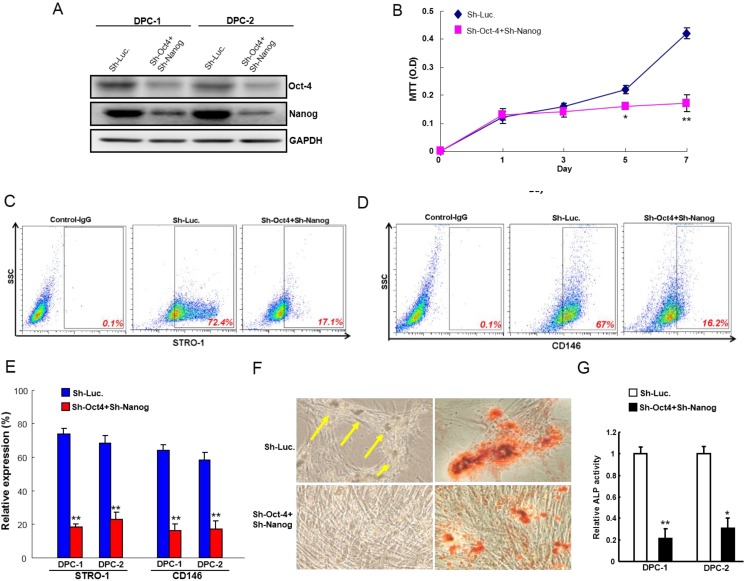
Depletion of *Oct4*/*Nanog* impairs proliferation rate and osteogenic properties in STRO-1^+^CD146^+^ DPSCs (**A**) Single cell suspensions of STRO-1^+^CD146^+^ DPSCs were transduced with sh-Luc or sh-*Oct4* + sh-*Nanog* lentivirus; Total proteins prepared from infected cells were prepared and analyzed. The silencing effect of *Oct4*/*Nanog* shRNA in DPSCs was validated translationally by western blotting; Immunoblotting against anti-Oct4, anti-Nanog, or anti-GAPDH antibodies was performed as indicated; The amount of GAPDH protein of different crude cell extracts was referred to as loading control and for further quantification; (**B**) The proliferation rate of sh-Luc or sh-*Oct4*/sh-*Nanog* STRO-1^+^CD146^+^ DPSCs was analyzed by a MTT assay; The expression of (**C**) STRO-1 and (**D**) CD146 of *Oct4*/*Nanog* co-knockdown DPSCs was analyzed by flow cytometry analysis; (**E**) The expression of STRO-1 and CD146 of *Oct4*/*Nanog* co-knockdown DPSCs was calculated and presented in the chart; (**F**) The osteogenic differentiation of DPSCs cultivated with osteogenic medium infected with sh-Luc or *Oct4*/*Nanog*-shRNA lentivirus was examined. Yellow arrow indicates mineralized nodule formation in DPSCs; (**G**) ALP activity of *Oct4*/*Nanog* co-knockdown DPSCs was analyzed. Data shown here are the mean ± SD of three independent experiments. (* *p* < 0.05; ** *p* < 0.01).

To further study the effect of *Oct4* and *Nanog* on MSC properties, we stably co-knocked down *Oct4* and *Nanog* expression in DPSCs. Immunoblotting analyses confirmed that lentivirus expressing both sh-*Oct4* and sh-*Nanog* markedly reduced the co-expression level of *Oct4* and *Nanog* protein in transduced STRO-1^+^CD146^+^ DPSCs (Figure 3A). The cell proliferation rate was also decreased in sh-*Oct4*/sh-*Nanog* infected DPSCs cultured in regular medium by MTT analysis (Figure 3B). Co-knockdown of *Oct4* and *Nanog* effectively inhibited the expression level of STRO-1 and CD146 in DPSCs (Figure 3C–E). DPSCs have been shown to exhibit the osteogenesis differentiation capacity [2]. Importantly, DPSCs infected with sh-*Oct4*/sh-*Nanog* expressing lentivirus significantly decreased the percentage of mineralized nodule formation and osteogenic differentiation potential when cultured with osteogenic medium (Figure 3F). Alkaline phosphatase is a well-known marker for for osteogenesis [2]. Co-targeting endogenous *Oct4* and *Nanog* expression dramatically decreased ALP activity in DPSCs (Figure 3G) when cultured with osteogenic medium. However, co-knockdown of *Oct4*/*Nanog* did not change osteogenic differentiation in DPSCs without osteoinduction media (data not shown). Together, *Oct4* and *Nanog* cooperatively modulated cell proliferation rate as well as MSCs properties of DPSCs.

### 2.4. Overexpression of Oct4/Nanog Enhanced Stemness and Proliferation Activity of DPCs

To investigate the effect of *Oct4* or *Nanog* up-regulation on biological properties of DPCs, we stably overexpressed *Oct4* or *Nanog* expression in DPCs through lentiviral-mediated transduction. Immunoblotting analyses confirmed that over-expression *Oct4* or *Nanog* markedly increased the expression level of *Oct4* (Figure S1A) or *Nanog* (Figure S1B) protein in transduced DPCs. Consistent with knockdown experiments, single overexpression of *Oct4* (Figure S1C) or *Nanog* (Figure S1D) expression did not affect the proliferation rate of DPCs. The expression of STRO-1 and CD146 in DPCs did not change in *Oct4* or *Nanog*-overexpressing DPCs (Figure S1D). Single over-expression of *Oct4* or *Nanog* was not capable of changing ALP activity (Figure S1E) in DPCs.

**Figure 4:**
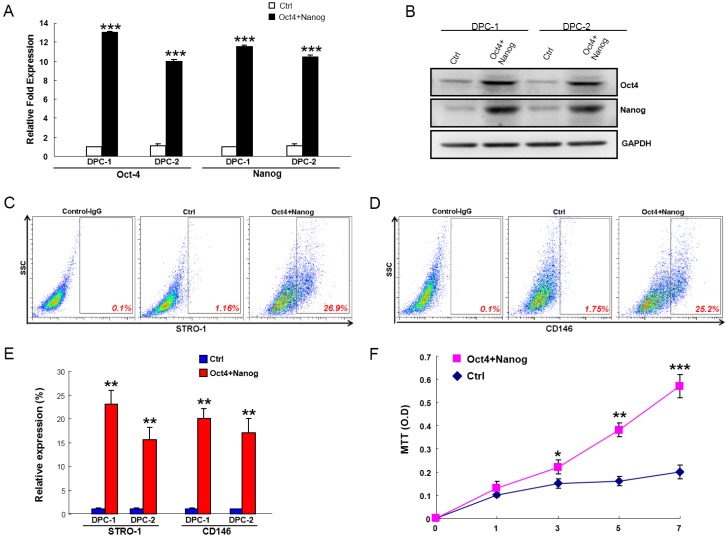
Overexpression of Oct4/Nanog in DPCs enhances STRO-1 expression and proliferation rate. Expression of *Oct4*/*Nanog* transcript (**A**) or protein (**B**) in DPCs infected with either *Oct4*/*Nanog*-overexpressing or control GFP lentiviruse were examined by real-time RT-PCR analysis (**A**) and western blot (**B**); The amount of GAPDH protein was referred to as the loading control; (**C**) STRO-1 and (**D**) CD146 expression in *Oct4*/*Nanog*-overexpressing or control-GFP DPCs was analyzed by flow cytometry; (**E**) STRO-1 and CD146 expression of *Oct4*/*Nanog* co-overexpressing DPCs were calculated and presented in the chart; (**F**) The proliferation rate of control-GFP and *Oct4*/*Nanog* overexpressing DPCs was analyzed by MTT assay. Data shown here are the mean ± SD of three independent experiments. (* *p* < 0.05; ** *p* < 0.01; *** *p* < 0.001).

To further evaluate the functional roles of *Oct4*/*Nanog* co-expression in DPCs, we co-overexpressed *Oct4* and *Nanog* expression in DPCs. As expected, overexpression of *Oct4* and *Nanog* increased the transcripts (Figure 4A) and protein levels (Figure 4B) in *Oct4*/*Nanog*-overexpressing DPCs. Notably, the endogenous STRO-1 (Figure 4C) and CD146 (Figure 4D) was enhanced by co-overexpressed *Oct4*/*Nanog* in DPCs (Figure 4E). Meanwhile, *Oct4*/*Nanog*-overexpressing DPCs cultivated with regular medium also showed significantly elevated proliferation rates in comparison to control DPCs by MTT analyses (Figure 4F). However, *Oct4*/*Nanog*-overexpressing DPCs also regulate the phenotypes comparison with wild type cells (data not shown).

### 2.5. Overexpression of Oct4/Nanog Increased Osteogenic/Chondrogenic/Adipogenic Properties of DPCs

**Figure 5:**
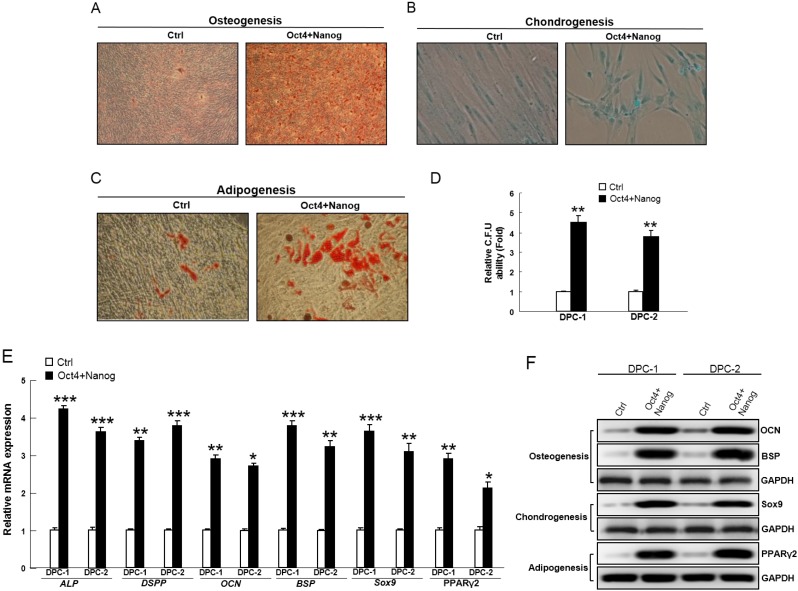
Overexpression of *Oct4*/*Nanog* enhanced osteogenic/chondrogenic/adipogenic induction differentiation properties in DPCs. Representative images of osteogenesis (**A**) and chondrogenesis (**B**) and adipogenesis (**C**) capability of control-GFP or *Oct4*/*Nanog*-overexpressing DPCs when cultured with osteogenic or chondrogenic or adipogenic induction differentiation medium; The representative image of triplicate samples from one representative experiment (**D**) Colony formation ability of control-GFP and *Oct4*/*Nanog*-overexpressing DPCs cultivated with regular medium was analyzed; (**E**) Transcript levels of osteogenic markers (ALP, DSPP, OCN, and BSP) and chondrogenic marker (Sox9) and adipogenic marker (PPARγ2) in control-GFP or *Oct4*/*Nanog*-overexpressing DPCs were analyzed by real-time RT-PCR analysis; (**F**) Protein levels of osteogenic markers (OCN and BSP) and chondrogenic marker (Sox9) and adipogenic marker (PPARγ2) in control-GFP or *Oct4*/*Nanog*-overexpressing DPCs were analyzed by western blotting analysis. Data shown here are the mean ± SD of three independent experiments. (* *p* < 0.05; ** *p* < 0.01; *** *p* < 0.001).

The co-overexpression of *Oct4*/*Nanog* in DPCs induces an increase of the STRO-1 and CD146 expression potential and in consequence an increase in cell proliferation rate and an increase of the DPCs pluripotency. These data suggested that the crucial role of *Oct4*/*Nanog* co-expression in the maintenance of MSCs characteristics of DPCs. We then evaluated *Oct4*/*Nanog* overexpression in DPCs under osteogenic and chondrogenic and adipogenic induction differentiation states. The proper differentiation of different lineages was induced by specific induction differentiation protocols described in the Materials and Methods section. Two weeks after the osteogenic induction, *Oct4*/*Nanog* overexpression significantly enhanced the osteogenic efficiency in DPCs cultured with osteogenic induction medium (Figure 5A). DPCs with *Oct4*/*Nanog* overexpression also showed an increase in chondrogenic induction differentiation (Figure 5B) and adipogenic induction differentiation (Figure 5C) potentials in DPCs when cultured with chondrogenic or adipogenic induction medium. Meanwhile, this *Oct4*/*Nanog* co-overexpression significantly increased the colony-forming efficiency in DPCs cultivated with regular medium (Figure 5D). The discovery of the association between expression of *Oct4*/*Nanog* and DPSCs suggests the possibility that *Oct4*/*Nanog* may regulate the osteogenic/chondrogenic/adipogenic differentiation markers. Quantitative RT-PCR showed up-regulation of osteogenic markers (ALP, DSPP, BSP, and OCN) and chondrogenic markers (Sox9) and adipogenic markers (PPARγ2) in *Oct4*/*Nanog*-overexpressing DPCs cultivated with specific induction differentiation medium (Figure 5E). Accordingly, the western blotting data showed that the proteins levels of BSP, OCN, Sox9, and PPARγ2 were also up-regulated in *Oct4*/*Nanog*-overexpressing DPCs cultivated with specific induction differentiation medium (Figure 5F). However, these differentiation markers did not change in *Oct4*/*Nanog*-overexpressing DPCs cultivated with regular medium (data not shown). Taken together, these data indicate that the overexpression of *Oct4*/*Nanog* caused DPC cells to enhance colony-forming efficiency and to promote the osteogenic/chondrogenic/adipogenic induction differentiation capability but not spontaneous differentiation.

## 3. Discussion

Dental pulp stem cells (DPSCs), which possess multipotent-differentiation capability, are a candidate for regenerative medicine [14]. They described the identification of DPSCs by virtue of their clonogenic abilities, rapid proliferative rates, and capacity to form mineralized tissues both *in vitro* and *in vivo.* Compared with other stem cells such as MSCs from the bone marrow and NSCs from cadavers, DPSC are more easily isolated [15]. However, preliminary studies have identified that the stem cell or potential progenitor cell population in dental pulp comprises less than 1% of the total cells [1,16]. Therefore, an improved comprehension of the cellular and molecular mechanisms that elevate stemness properties of DPSCs could be pursued to bring forth future progress in regenerative medicine.

Self-renewal and pluripotency are the central features in the definition of ESCs, in which *Oct4* and *Nanog* play a key role on maintenance of the process [8]. However, *Oct4*/*Nanog* mediated molecular mechanisms in DPSCs still remain to be elucidated. Herein, we evaluated the role of *Oct4*/*Nanog* in the maintenance of MSCs characteristics of DPCs by lentiviral-mediated co-knockdown or co-overexpression of *Oct4* and *Nanog*. Depletion of *Oct4*/*Nanog* decreased proliferation and osteogenic properties of DPSCs (Figure 2). Co-overexpression of *Oct4*/*Nanog* enhanced the proliferation rate and osteogenesis/chondrogeneisis/adipogenesis properties of DPCs (Figure 5). Collectively, our data first demonstrated the crucial role of *Oct4*/*Nanog* co-expression in the MSCs properties enhancement of dental pulp cells.

*Oct4* and *Nanog* are also the key transcription factors that could reprogram a human somatic fibroblast into an embryonic stem cell-like pluripotent cell, termed inducible pluripotent stem cell (iPSC) [13]. The dental pulp tissue can be isolated from human exfoliated deciduous and permanent teeth which are usually regarded as medical waste [17]. Previous studies have shown that the DPCs including DPSCs express elevated levels of pluripotent factors, resulting in significantly more efficient and accelerated generation of induced pluripotent stem cells compared with conventional somatic cells. The c-Myc oncogene may contribute to tumorigenesis by over stimulating cell growth and metabolism and/or by causing genomic instability. Deregulated expression of c-Myc occurs in a wide range of human cancers and is often associated with poor prognosis, indicating a key role played by this oncogene in tumor progression. Previous studies have successfully generated iPSCs from mouse and human fibroblasts using only three transcription factors: Oct-4/Sox2/Klf4 (without c-Myc), and mice derived from these iPSCs without c-Myc did not develop tumors during the study period [18,19]. In the present study, co-overexpression of *Oct4*/*Nanog* enhanced the proliferation rate and MSCs properties of DPCs (Figure 4). Further research on reprogramming human DPCs using *Oct4*/*Nanog* without c-Myc from exfoliated deciduous and permanent teeth into iPSCs for personalized therapies for human disease is needed.

Dnmt1, a major DNA methyltransferase, is responsible for maintenance of the DNA methylation pattern during DNA replication [20]. DNA methylation is crucial for embryo development or ESCs differentiation [21]. Tsai et al reported that *Oct4* and *Nanog* cooperatively modulate the proliferative and undifferentiated states of bone marrow stem cells (BMSCs) by directly binding to the promoter region of Dnmt1 [22]. BMSCs treated with an inhibitor of DNA methylation or transfected Dnmt1 siRNA attenuated proliferation rate and differentiation potential [22]. Epithelial-mesenchymal transition (EMT), a transdifferentiation program that converts epithelial state into mesenchymal state, is critical for development and disease [23]. Intensive studies revealed that transcriptional factors, such as Snail, Slug, and Twist regulate the EMT process [24]. Slug, the best-characterized member of the Snail superfamily, regulate chondrogenic phenotypes in MSCs [25]. In lung cancer cells, co-overexpression of *Oct4* and *Nanog* enhanced the tumor-initiating capability through activation of Slug expression [26]. Therefore, it would be interesting to know whether the regulation of Dnmt1 or EMT by *Oct4* and *Nanog* is also found in DPSCs.

In cancer cells, aberrant expression of *Oct4* or *Nanog* has been linked to an increased proliferation rate *in vitro* and *in vivo* [27,28]. Knockdown of *Nanog* alone in breast cancer cells presented suppressed clonogenic growth and proliferation [29]. A global gene expression profile screening in *Nanog* small interfering RNA (siRNA)-transfected embryonal carcinoma cells suggested the involvement of *Nanog* in a cell cycle-signaling pathway [30]. This group reported that along with *Nanog* knockdown, several cell cycle-related genes as well as a p53-related signaling pathway were downregulated, suggesting a role of *Nanog* in cell cycle regulation and survival [30]. In breast cancer cells, Han and colleagues found that knockdown of Nanog resulted in inhibited cell proliferation, G0/G1 arrest of the cell cycle, and suppressed expression of cyclin D1 and oncogene c-Myc [29]. They further demonstrated that *Nanog* directly binds to the minimal promoter region of cyclin D1, transcriptionally regulates its expression, and thus drives the cell cycle moving forward and accelerates cell proliferation [29]. However, the existence of the regulation, as well as its physiological effects, in DPSCs is still elusive. Further exploration of the mechanisms through which *Oct4*/*Nanog* regulates cell proliferation is still needed to shed light on the role of *Oct4*/*Nanog* in MSCs properties.

In ESCs, *Nanog* is involved in a complicated stemness regulatory network in cooperation with other key transcriptional factors, such as Oct, Sox2, and Lin 28, to precisely balance between pluripotency and differentiation tendency [31,32]. Kalmar and colleagues reported that *Nanog* presents a transcriptional fluctuation between low and high expression level in ES cells [33]. They proposed that the dynamic distribution of *Nanog* is crucial for ESCs to maintain the undifferentiated state and yet possess the potential to be differentiated without marking definitive commitment if proper differentiation signaling strikes the cells [33]. The tightly regulated *Nanog* seems to be a key switch that determines the fate of ESCs [34]. Accumulating reports have recently placed interests on identifying stemness-related signaling pathways, and miRNAs, in hope of identifying targets for potential regenerative medicine. Through targeting the core stemness factors such as *Oct4*, *Nanog*, and Sox2, the up- and down-regulation of miRNAs including miR134, miR296, and miR470, plays roles in modulating the self-renewal, pluripotency, and differentiation of ESCs [35]. miR-134 was first found directly targeting *Nanog* in mouse ESCs, through which it promotes mouse ES cell differentiation toward ectodermal lineages [36]. Further research effort on elucidating the mechanisms by which *Oct4*/*Nanog* factors regulated by miRNAs in DPSCs is needed.

## 4. Experimental Section

### 4.1. Cultivation of Primary Cells from Dental Pulps Tissues

Dental pulp tissues were freshly derived from a caries-free intact premolar that was extracted for orthodontic treatment purpose. All of the clinical samples in this study were collected after obtaining written informed consent and this study was reviewed and approved by the Institutional Review Board in Chung Shan Medical University Hospital (CSMUH No.: CSI13108). Dental pulp tissue was immersed in phosphate buffered saline (Caisson, North Logan, UT, USA) solution and enzymatically dissociated into single cells. These cells were grown in Dulbecco’s Modified Eagle Medium (DMEM) supplemented with 10% FBS, 2 mM l-glutamine, 100 U/mL penicillin-G and 100 μg/mL streptomycin. (GIBCO BRL, Grand Island, NY, USA).

### 4.2. Identification of Cell Phenotypic Markers by FACS

Dental pulp cells were used for phenotypic marker identification by flow cytometry. 10^5^ cells were resuspended in 100 μL PBS and incubated with primary STRO-1 and CD146 antibodies at 4 °C for 1 h with 1:100 dilutions. The labeled cells were suspended in 100 μL PBS with 1 μL goat anti-mouse IgG conjugated with FITC (Chemicon Inc., Temicula, CA, USA) at 4 °C for 1 h, then examined with a FACSCalibur apparatus (Becton Dickinson, La Jolla, CA, USA)

### 4.3. Construction of Lentiviral-Mediated RNAi for Silencing Oct4 and Nanog

The pLV-RNAi vector, which co-expressed for GFP protein in infected host cells, was purchased from Biosettia Inc. (Biosettia, San Diego, CA, USA). The method of cloning the double-stranded shRNA sequence was described in the manufacturer’s protocol. Lentiviral vectors expressing shRNA that target human *Oct4* or *Nanog* (oligonucleotide sequence: sh-*Oct4*: 5'-AAAAGCTTCAAGAACATGTGTAATTGGATCCAATTACACATGTTCTTGAAGC-3'; sh-*Nanog*: 5'-AAAAGCATCCGACTGTAAAGAATTTGGATCCAAATTCTTTACAGTCGGATGC-3') were synthesized and cloned into pLVRNAi to generate a lentiviral expression vector. sh-Luc: 5'-CCGGACTTACGCTGAGTACTTCGAACTCGAGTTCGAAGTACTCAGCGTAAGTTTTTTG-3' was utilized for an experimental control. Lentivirus production was performed by co-transfection of plasmid DNA mixture with lentivector plus helper plasmids (VSVG and Gag-Pol) into 293T cells (American Type Culture Collection, Manassas, VA, USA) using Lipofectamine 2000 (LF2000, Invitrogen, Calsbad, CA, USA). The lentivirus multiplicity of infection (MOI) titer was determined by flow cytometry (average of 5 × 10^4^ and 2 × 10^5^ TU/mL). The green fluorescence protein (GFP), which was co-expressed in lentiviral-infected cells, served as a selection marker to indicate the successfully infected DPC cells.

### 4.4. Stable Overexpression of Oct4 and Nanog in DPC Cells (DPCs)

Human *Oct4* and *Nanog* cDNA template were cloned into pCDH1-MCS1-EF1-copGFP (System Biosciences, Cat. No.: CD511A-1; Mountain View, CA, USA). Lentivirus production was performed as above. Stable *Oct4*/*Nanog*-overexpressing DPC cells were further purified by cell sorting with GFP positive cells. The pCDH1-MCS1-EF1-copGFP empty vector alone was utilized for the experimental control.

### 4.5. Quantitative Real-Time Reverse-Transcriptase (RT)-PCR

Briefly, total RNA (1 μg) of each sample was reverse-transcribed in a 20-μL reaction using 0.5 μg oligo(dT) and 200 U Superscript II RT (Invitrogen, Carlsbad, CA, USA). The amplification was carried out in a total volume of 20 μL containing 0.5 μM of each primer, 4 mM MgCl_2_, 2 ΜL LightCycler™–FastStart DNA Master SYBR green I (Roche Molecular Systems, Alameda, CA, USA) and 2 μL of 1:10 diluted cDNA. PCR reactions were prepared in duplicate and heated to 95 °C for 10 min, followed by 40 cycles of denaturation at 95 °C for 10 s, annealing at 55 °C for 5 s, and extension at 72 °C for 20 s. Standard curves (cycle threshold values *versus* template concentration) were prepared for each target gene and for the endogenous reference (GAPDH) in each sample. Quantification of unknown samples was performed using LightCycler Relative Quantification Software version 3.3 (Roche Molecular Systems). Primer sequences are listed in Table 1.

**Table 1:** The sequences of the primers for quantitative RT-PCR.

Gene (Accession No.)	Primer Sequence (5' to 3')	Product Size (bp)	Tm (°C)
*Oct4* (NM_002701)	F: GTGGAGAGCAACTCCGATG	86	60
	R: TGCTCCAGCTTCTCCTTCTC		
*Nanog* (NM_024865)	F: ATTCAGGACAGCCCTGATTCTTC	76	60
	R: TTTTTGCGACACTCTTCTCTGC		
*Sox2* (NM_003106)	F: GACTTCACATGTCCCAGCACTA	156	60
	R: CTCTTTTGCACCCCTCCCATT		
*ALP* (NM_000478)	F: CCACGTCTTCACATTTGGTG	99	60
	R: ATGGCAGTGAAGGGCTTCTT		
*DSPP* (NM_014208)	F: TCACAAGGGAGAAGGGAATG	187	60
	R: CTGGATGCCATTTGCTGTGA		
*OCN* (NM_199173)	F: GGCAGCGAGGTAGTGAAGAG	160	60
	R: GCCGATAGGCCTCCTGAAAG		
*BSP* (NM_004967)	F: AAAGTGAGAACGGGGAACCT	95	60
	R: ACCATCATAGCCATCGTAGCC		
*GAPDH* (NM_002046)	F: CATCATCCCTGCCTCTACTG	180	60
	R: GCCTGCTTCACCACCTTC		

### 4.6. Western Blot Assay

The extraction of proteins from cells and immunoblotting analysis were performed. Fifteen microliters of sample was boiled at 95 °C for 5 min and separated on 10% acrylamide gels for SDS-PAGE. The proteins were transferred to Hybond-ECL nitrocellulose paper or PVDF membrane (Amersham, Arlington Heights, IL, USA) by a wet-transfer system. The reactive protein bands were detected by the ECL detection system (Amersham).

### 4.7. Assays for Cell Proliferation 

An MTT assay kit (Sigma-Aldrich, Oakville, ON, Canada) was used to analyze the cell proliferation. Specifically, 1 × 10^3^ cells were seeded in each well of a 24-well plate, and then 10 μL of MTT solution was added to the cells which were then incubated at 37 °C for 3 h. The supernatant was removed, and 200 μL of DMSO were added directly to the cells. The MTT color reaction was analyzed using a microplate reader set at *A*560 nm.

### 4.8. Alkaline Phosphatase Activity (ALP)

For detecting the ALP activity of cells on original plates, cells were fixed with 80% alcohol, and then fixed cells were stained using the Vector Blue Alkaline Phosphatase Substrate Kit III (Vector Laboratories Inc., Burlingame, CA, USA) according to the manufacturer’s instructions.

### 4.9. In Vitro Osteogenic Differentiation

Cells were cultured in DMEM-LG (Invitrogen) supplemented with 15% FBS, 50 μg/mL ascorbate-2-phosphate, 10 nmol/L dexamethasone, and 10 mmol/L β-glycerophosphate (Sigma, St. Louis, MO, USA) for 2 weeks. At the end of osteogenic induction, cells were washed twice with PBS, fixed for 10 min at room temperature with 3.7% paraformaldehyde, and stained with Alizarin red to assess osteogenic differentiation.

### 4.10. In Vitro Chondrogenic Differentiation

Cells were cultured in DMEM High Glucose supplemented with ITS + Premix: 6.25 μg/mL insulin, 6.25 μg/mL transferrin, 5.33 μg/mL linoleic acid, 1.25 μg/mL bovine serum albumin, 10^−7^ M dexamethasone, 50 μg/mL ascorbate-2 phosphate, 1 μM sodium pyruvate, 100 μg/mL penicillin and 10 μg/mL streptomycin) (Sigma) in the presence of TGF-β1 (10 ng/mL) (Mylteny, Bergisch, Gladbach, Germany) for 2 weeks. At the end of chondrogenic induction, cells were washed twice with PBS, fixed for 10 min at room temperature with 3.7% paraformaldehyde, and stained with Alcian Blue to assess chondrogenic differentiation.

### 4.11. In Vitro Adipogenic Differentiation

Cells were cultured in α-MEM supplemented with 16.6% FBS, 50 μg/mL ascorbate-2 phosphate, 10^−7^ M dexamethasone, 50 μM indomethacin, 0.45 mM 3-isobutyl-1-methyl-xanthine and 10 μg/mL insulin for 2 weeks. At the end of adipogenic induction, cells were washed twice with PBS, fixed for 10 min at room temperature with 3.7% paraformaldehyde, and stained with Oil Red O to assess adipogenic differentiation.

### 4.12. Statistical Analysis

The statistical package of Social Sciences software (version 13.0) (SPSS, Inc., Chicago, IL, USA) was used for statistical analysis. Student’s *t* test was used to determine the statistical significance of differences between experimental groups; *p* values less than 0.05 were considered statistically significant.

## 5. Conclusions

*Oct4* and *Nanog* may act as the major regulators in the maintenance of MSCs properties in DPSCs. After receiving proper differentiation induction, these *Oct4*/*Nanog* overexpressing DPCs increase osteogenic/chondrogenic/adipogenic properties that highly expressed various osteogenic/chondrogenic/adipogenic differentiation markers. Our findings may provide an alternative cell source and strategy for personalized therapies for human dental disease. Further studies to identify the cellular functions and molecular mechanisms of *Oct4* and *Nanog* in DPSCs are critical to have a better insight into their potential contribution to regenerative medicine.

## Supplementary Materials

Supplementary figure can be found at http://www.mdpi.com/1422-0067/15/10/18623/s1.

## Supplementary Files

Supplementary File 1
