# Supplementary material for: Concurrent Expression of Oct4 and Nanog Maintains Mesenchymal Stem-Like Property of Human Dental Pulp Cells
Source: Int J Mol Sci. 2014 Oct 15;15(10):18623–39. doi: 10.3390/ijms151018623 (PMC4227236; doi:10.3390/ijms151018623)

Supplementary Information

Figure S1. Single over-expression of *Oct4* or *Nanog* expression did not alter proliferation rate and ALP activity in DPCs. The over-expression effect of *Oct4* (**A**) or *Nanog* (**B**) DPCs was validated translationally by western blotting. Immunoblotting against anti-Oct4, anti-Nanog, or anti-GAPDH antibodies was performed as indicated. The amount of GAPDH protein of different crude cell extracts was referred to as loading control and for further quantification; The proliferation rate of *Oct4* (**C**) or *Nanog* (**D**) over-expression in DPCs was analyzed by a MTT assay; (**E**) The expression of STRO-1 and CD146 of *Oct4* or *Nanog*-overexpressing DPCs was analyzed by flow cytometry analysis; (**F**) ALP activity of *Oct4* or *Nanog*-overexpressing DPCs was analyzed. Data shown here are the mean ± SD of three independent experiments.

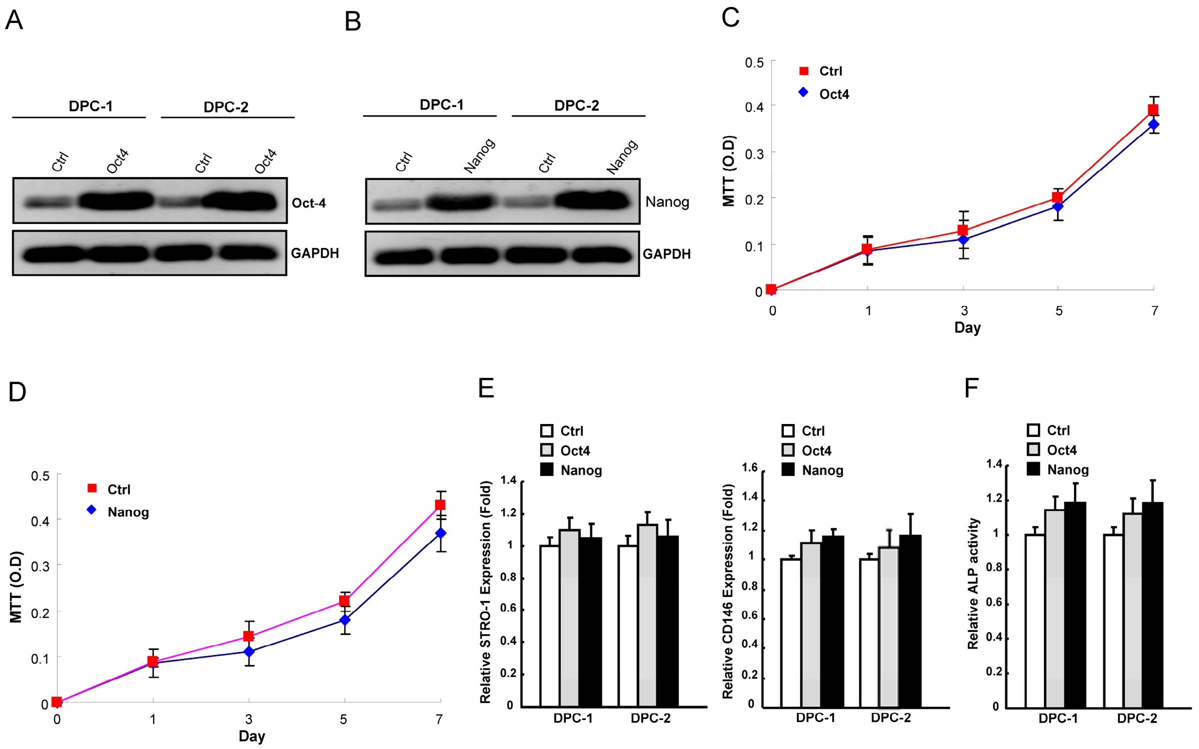

Supplement: Supplementary File 1 [file ijms-15-18623-s001.pdf]
